# Tympanic membrane perforation caused by traumatic asphyxia

**DOI:** 10.5935/1808-8694.20130023

**Published:** 2015-10-14

**Authors:** Fernando Luiz Westphal, Renato Telles de Sousa, Luiz Carlos Nadaf de Lima, Luís Carlos de Lima, Márcia dos Santos da Silva

**Affiliations:** Post-doctoral degree in Thoracic Surgery (Coordinator of Educaton and Research at the Getúlio Vargas University Hospital); MSc in ENT (Head of the ENT Service at the Getúlio Vargas University Hospital); MSc in ENT (Assistant Physician at the Getúlio Vargas University Hospital); PhD in Heart Surgery (Head of the Thoracic Surgery Service at the Getúlio Vargas University Hospital); ENT Resident Physician (Fist-year ENT Resident Physician). Getúlio Vargas University Hospital

**Keywords:** hearing loss, thoracic injuries, tinnitus, tympanic membrane perforation

## INTRODUCTION

Tympanic membrane (TM) trauma may be caused by excessive pressure in the middle ear, thermal or chemical burns, direct penetrating trauma, or barotrauma. Increased pressure outside the ear is, by far, the most frequent mechanism by which TM trauma occurs^1^. This paper describes an unusual case of TM perforation caused by increased venous pressure in the head and neck area after acute chest trauma.

## CASE PRESENTATION

A 31-year-old male subject had his chest compressed by a mechanical arm while he was working at an industrial plant. The subject lost his consciousness temporarily at the time of the accident and was sent to an Intensive Care Unit complaining of dyspnea, intense chest pain, and bilateral otalgia and tinnitus.

**Figure 1:**
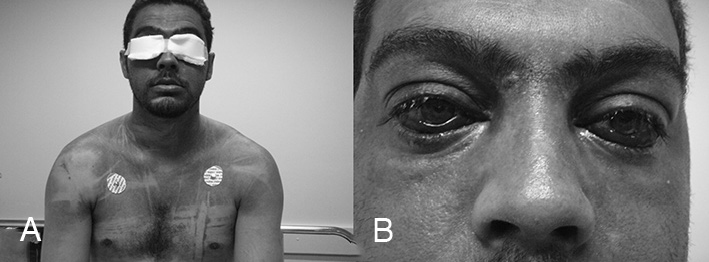
A: Cyanosis of the upper third of the chest extending to the neck and head. B: Bilateral subconjunctival hemorrhage.

Physical examination: cyanosis of the face, upper third of the chest, and anterior and posterior portion of the chest (Figure 1A); bilateral otorrhagia and epistaxis, and subconjunctival hemorrhage (Figure 1B). Consciousness, respiratory and heart rates were normal. Cardiopulmonary auscultation, abdomen and lower limb examination did not reveal alterations. Otoscopic examination: bilateral TM perforation; perforation located centrally and affecting approximately 25% of the TM area on both sides.

Complementary tests: echocardio-gram was normal; chest CT scans showed minor left pleural effusion and two fractured costal arches. Lab tests revealed CPK-MB of 2540 IU and normal troponin levels. Audiometry tests were not available at admission.

The patient remained in the ICU for five days and for an additional six in the infirmary. He progressed well and his symptoms gradually subsided. Audiometry tests were carried out eight days after admission and the patient was found to have mild left conductive hearing loss and auditory thresholds within normal range in his right ear. Otoscopic examination was performed 20 days after the accident and showed signs of formation of a new tympanic membrane on the sites where the TM had been bilaterally perforated. Another audiometric examination was carried out 30 days after the trauma and showed normal thresholds on both ears.

## DISCUSSION

Traumatic asphyxia (TA), also known as ecchymotic mask or Perthes syndrome, is a rare condition characterized by head and neck cyanosis, subconjunctival hemorrhage, and head and neck petechiae^2^. In order for the syndrome to occur, subjects have to undergo chest or chest and abdomen compression after inhaling with the glottis closed^3^. The sharp increase in intrathoracic pressure forces the blood on the right side of the heart and large chest veins to be ejected abruptly towards the head, producing blood stasis and rupturing capillaries. Thus, in addition to classic symptoms, there may also be varying degrees of mouth, ear, and nose bleeds, accompanied by tinnitus and transient hearing loss^4^. In the presented case, venous hypertension reached levels high enough to induce the rupturing of the TM. The patient complained of otalgia and tinnitus - symptoms compatible with his clinical status - but had no hypacusis.

It has been established that most traumatic TM perforations heal spontaneously. In large centers, patients in such condition are placed under observation. Factors such as secondary infection, pre-existing tympa-nosclerosis, and size and site of perforation are the main prognostic factor for healing^5^. Surgery is indicated only if the TM does not heal within three to six months. Studies suggest that 70% of central perforations, as observed in this patient, heal spontaneously^6^. The patient evolved satisfactorily. His TM was completely healed after 30 days and he had no signs of remaining hearing loss.

## CLOSING REMARKS

Many are the etiologies of traumatic TM perforation, and unusual cases may occur as reported in this case. Victims of compression trauma who develop Perthes syndrome require ENT care to avoid the late complications associated with TM perforation.
